# Influence of environmental exposures on T follicular helper cell function and implications on immunity: a comparison of Bangladeshi and American children

**DOI:** 10.1128/mbio.03980-24

**Published:** 2025-03-10

**Authors:** Dana M. Van Fossen, Hyunjae Cho, Lisa E. Wagar, Jennie Z. Ma, Julie Parsonnet, Rashidul Haque, Mark M. Davis, William A. Petri

**Affiliations:** 1 Division of Infectious Diseases and International Health, University of Virginia School of Medicine, Charlottesville, Virginia, USA; 2 Department of Public Health Sciences, University of Virginia School of Medicine, Charlottesville, USA; 3 Department of Physiology and Biophysics, University of California, Irvine, Irvine, California, USA; 4 Department of Epidemiology and Population Health, Stanford University, Stanford, California, USA; 5 Infectious Diseases Division, International Centre for Diarrheal Disease Research, Bangladesh, Dhaka, Bangladesh; 6 Department of Microbiology and Immunology, Stanford University School of Medicine, Stanford, California, USA; University of Wisconsin-Madison, Madison, Wisconsin, USA

**Keywords:** T follicular helper cells, immune dysfunction, malnutrition, childhood immunity, antibiotic exposure, T cell exhaustion

## Abstract

T follicular helper (Tfh) cells are crucial for B cell activation and subsequent antibody production. This functionality is influenced by surface markers such as CD40L, a costimulatory factor which promotes B cell activation, and CD57, which is a well-known marker of senescence. This study examined age-specific differences in Tfh cell function in Bangladeshi and American children. At age two, Bangladeshi children displayed impaired CD40L upregulation and significant CD57 downregulation upon stimulation. These patterns, not observed in American children of the same age, suggested an exhaustion-like phenotype potentially driven by environmental factors. Random forest and generalized estimating equations (GEE) modeling was used to analyze predictors of Tfh cell response to stimulation. Days since the last antibiotic treatment, total antibiotic treatments, diarrheal episodes, and malnutrition were identified as variables that significantly impacted the Tfh response to stimuli. To assess Tfh cell ability to promote antibody responses, we correlated Tfh functionality with antibody concentration post-vaccination and in response to infection with *Cryptosporidium*, an endemic apicomplexan parasite. Increased CD40L expression upon stimulation correlated positively with anti-Poliovirus type 2/3 neutralizing antibody and anti-Cp17 (a *Cryptosporidium* sporozoite antigen) IgA concentrations. In contrast, increased CD57 expression was significantly correlated with decreased anti-Cp17 IgA. This indicates that an activation-supportive phenotype (CD40L+) may be more effective in promoting immunity than a senescent phenotype (CD57+). Together, these findings suggest that early-life environmental exposures may program Tfh cell functionality, impacting immune response potential in settings with high pathogen exposure.

**IMPORTANCE:**

T follicular helper (Tfh) cells are upstream mediators that shape the humoral immune response to specific antigens. The generation of an effective memory response to infection is vital to prevent subsequent reinfections. However, in areas with high burdens of exposure to infections, such as the urban community from Bangladesh studied here, children are consistently exposed to inflammatory pathogens. Specific environmental exposures significantly influenced Tfh cell activation and senescence phenotypes. Additionally, Tfh cell responses correlated with antibody concentrations following vaccination or infection, indicating that environmental factors may play a critical role in shaping effective immunity in early childhood.

## INTRODUCTION

T follicular helper (Tfh) cells, a subset of CD4+ T cells (identified as CXCR5+, PD-1+), play a pivotal role in the activation of B cells, leading to class switching and somatic hypermutation, resulting in high-affinity antibody production (1–3). As such, Tfh cell functionality is a key component of immune competency, particularly in early childhood when the immune system is developing and adapting to first-time environmental exposures and mounting protective responses after vaccination (4, 5).

The functional capacity of Tfh cells is, in part, regulated by surface markers associated with activation and senescence. CD40L, a costimulatory molecule, interacts with CD40 on B cells, promoting B cell proliferation and differentiation into antibody-producing cells and is commonly used as a marker of activation (6–9). Conversely, CD57 is associated with cellular senescence and terminal differentiation, a marker of limited proliferative potential and an increased susceptibility to apoptosis (10, 11). These markers represent two ends of the Tfh cell functional spectrum, with CD40L indicating active, stimulatory capability and CD57 denoting cellular aging and senescence.

We previously found that the T cells of children in Bangladesh (an area of high microbial exposure) had increased effector and senescence-like phenotypes, which were not seen in age- and sex-matched children located in America (an area of low microbial exposure). This attribute was also associated with environmental factors such as stunting (12). However, the impact of specific environmental factors has yet to be studied in the context of T follicular helper cell function. This study investigates age-specific differences in Tfh cell CD40L and CD57 expression and subsequent humoral response post stimulation.

Environmental exposures, including infection, nutritional status, and antibiotic usage, are known to shape immune development, particularly in early childhood (13–15). To understand how these factors influence Tfh cell functionality, we used random forest to identify important feature variables that influence cell responders. The covariates selected through random forest feature importance evaluation were used to fit generalized estimating equations (GEE) to assess the predictive power of specific environmental variables on CD40L and CD57 expression changes in Bangladeshi children. Furthermore, the functional state of Tfh cells has implications for immune outcomes, as these cells are integral to generating effective antibody responses post-vaccination or infection. To examine this outcome, we correlated CD40L and CD57 expression levels with antibody responses to routine vaccinations and *Cryptosporidium* infection, an endemic apicomplexan parasite in Bangladesh.

## RESULTS

### T follicular helper cells demonstrate age-specific changes in functional marker expression in Bangladeshi children at age 2

**Fig 1:**
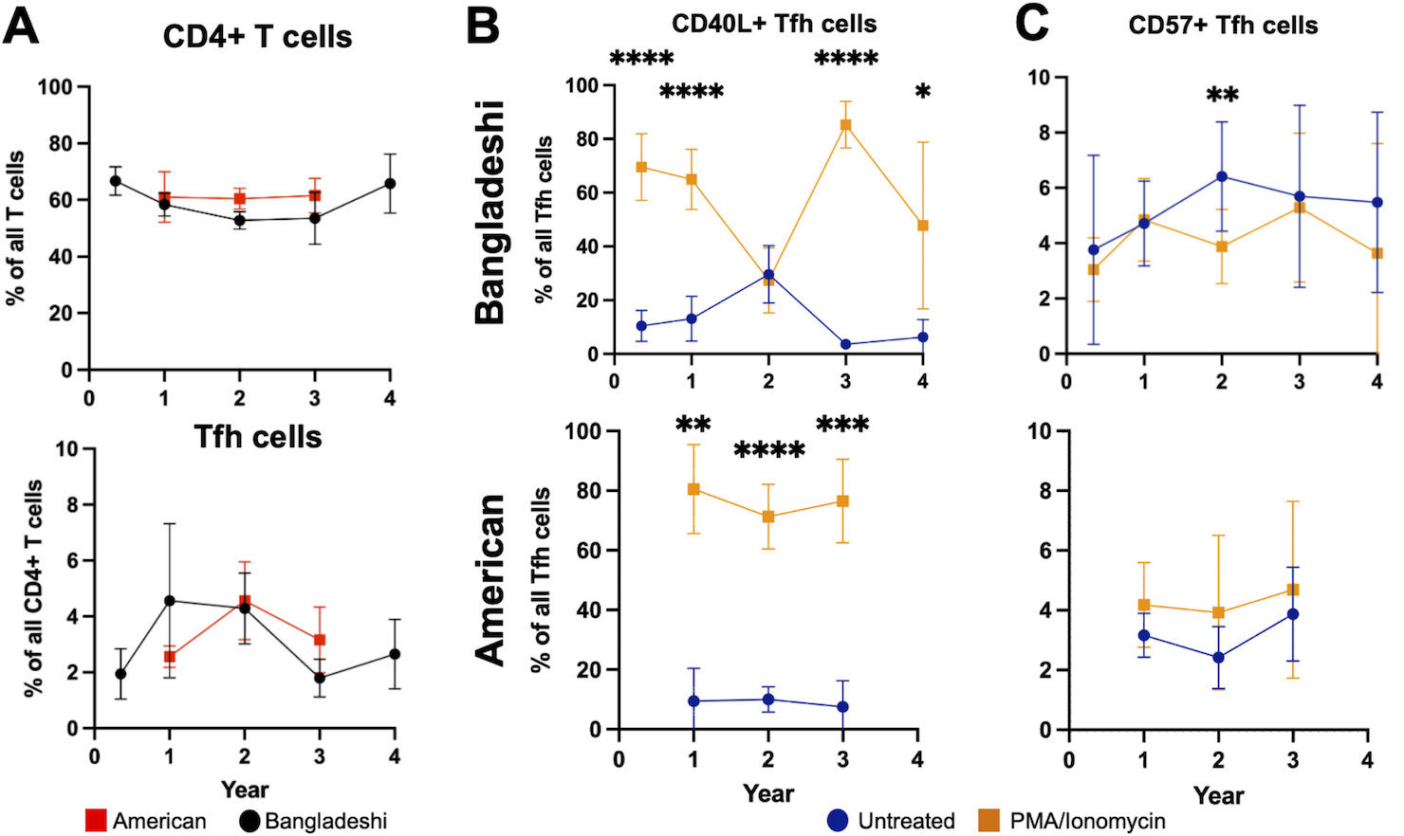
Comparison of CD4+ T cell and T follicular helper (Tfh) cell populations and function marker expression in American and Bangladeshi children. (**A**) Percentage of CD4+ T cells among all T cells and Tfh cells (CXCR5+, PD-1+) among all CD4+ T cells in American (red) and Bangladeshi (black) children across different ages. (**B**) Percentage of CD40L+ Tfh cells out of all Tfh cells, comparing untreated (blue) and PMA/Ionomycin-stimulated (orange) conditions. (**C**) Percentage of CD57+ Tfh cells out of all Tfh cells, comparing untreated (blue) and PMA/Ionomycin-stimulated (orange) conditions. (**B, C**) Bangladeshi samples were displayed in the top panel and American samples in the bottom panel. Data for each age represent cross-sectional analysis of subsets of children rather than paired samples from the same individuals across time points. Error bars represent confidence intervals. Statistical significance was calculated by multiple paired *t*-test corrected for multiple comparisons using the Benjamini-Krieger-Yekutieli method and denoted by asterisks (*P < 0.05, **P < 0.01, ***P < 0.001, ****P < 0.0001).

In both American and Bangladeshi children, the overall frequencies of CD4+ T cells and Tfh cells were comparable, suggesting that population-level differences in total T cell populations were minimal (Fig. 1A) and aligned with previous findings (12). However, significant differences in the expression patterns of CD40L and CD57 emerged in Bangladeshi children by age two, pointing to functional divergences in Tfh cell maturation or responsiveness. At age two, Bangladeshi children exhibited a diminished ability to upregulate CD40L following PMA/ionomycin stimulation, a phenotype not observed in their American counterparts or at other ages (Fig. 1B; Fig. S1). This lack of CD40L upregulation signified a potentially reduced functional capacity of Tfh cells in supporting B cell responses. Additionally, Bangladeshi children at age 2 showed a significant decrease in CD57 expression upon stimulation, in contrast to American children, who did not exhibit this pattern (Fig. 1C), suggesting an accelerated apoptotic process in these cells. Collectively, these findings indicated that at age 2, Bangladeshi children exhibited altered Tfh cell functionality, resembling an exhaustion-like phenotype potentially driven by environmental factors. This distinct immune profile was not observed in American children and may have implications for understanding immune competency across different environmental contexts.

To assess the potential for biased withdrawal of the sickest children, we compared malnutrition scores (WAZ, HAZ, and WHZ) at the time of termination across all five termination categories, including voluntary withdrawal, relocation, and death (Fig. S2) Statistical analysis by one-way ANOVA revealed no significant differences in WAZ (*P* = 0.1781), HAZ (*P* = 0.0525), or WHZ (*P* = 0.8280) scores between groups.

### Environmental factors predict expression changes of functional markers on T follicular helper cells upon stimulation, in Bangladeshi children at age 2

**Fig 2:**
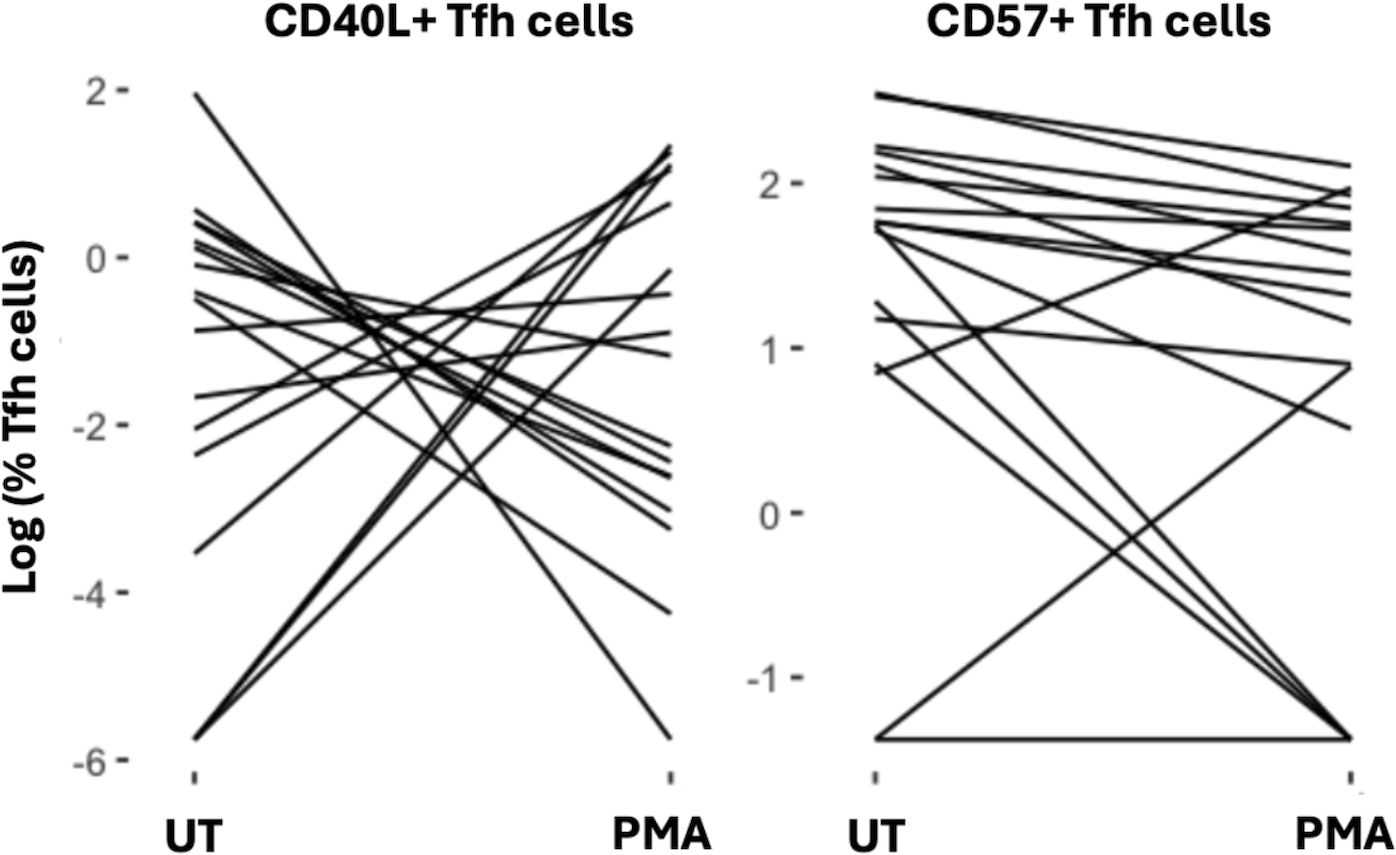
Variability in CD40L and CD57 expression on Tfh cells in response to stimulation in Bangladeshi children, at age 2. Spaghetti plots show log-transformed percentages of CD40L+ (left) and CD57+ (right) Tfh cells before (UT) and after (PMA) stimulation with PMA/Ionomycin in individual Bangladeshi children, at age 2.

The variability in CD40L and CD57 expression on Tfh cells in Bangladeshi children, at age 2, underscored the potential influence of environmental factors on immune cell functionality. Noteworthy fluctuations in the expression of CD40L on Tfh cells upon stimulation showed a diverse functional response within this population at age 2 (Fig. 2). To identify specific environmental factors associated with these functional changes, we applied random forest feature importance evaluation and generalized estimating equations (GEE) to assess predictive associations. The models evaluated how variables such as nutrition, infections, and antibiotic treatments influenced Tfh cell response to stimulation. Functional response to PMA/Ionomycin stimulation was measured by the ratio of percent CD40L+ or CD57+ Tfh cells post-stimulation to pre-stimulation levels.

**Fig 3:**
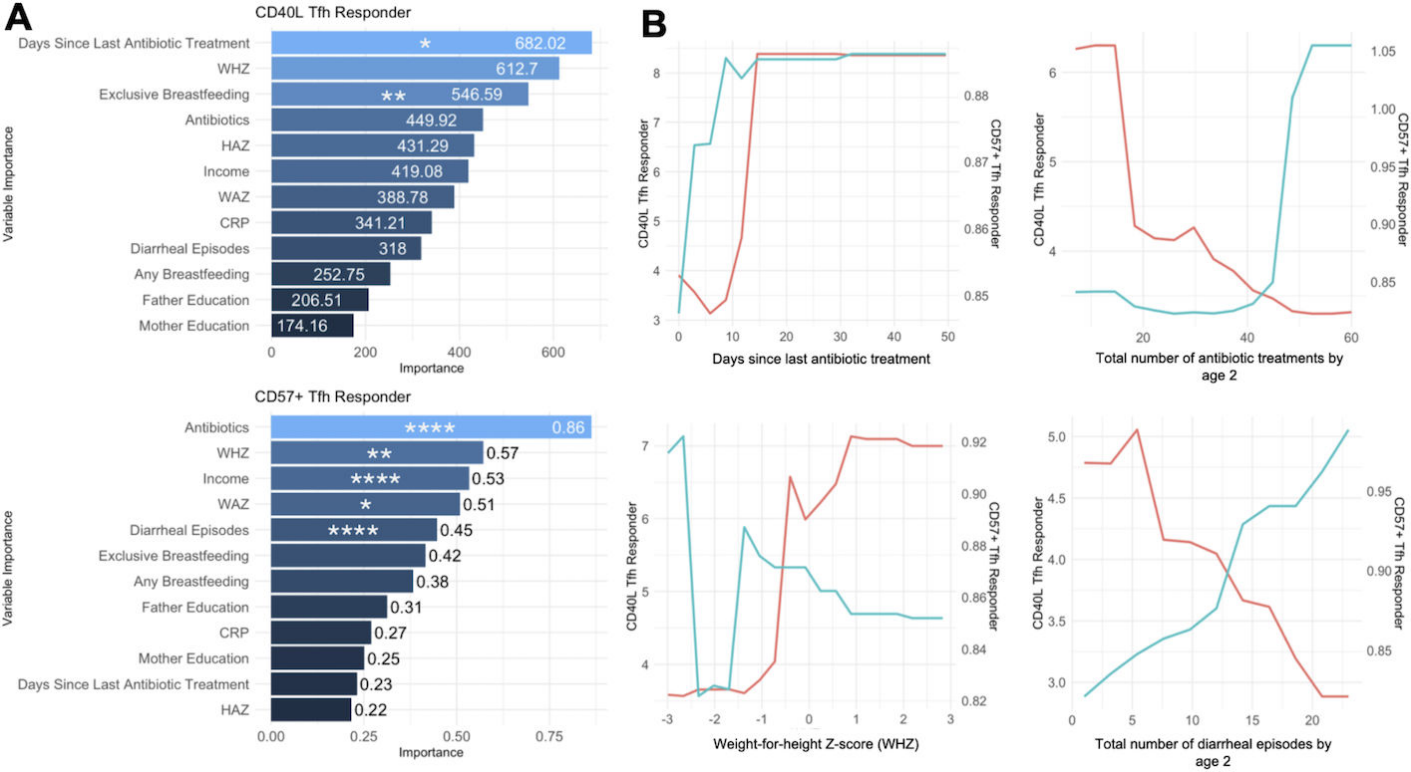
Environmental predictors of CD40L and CD57 expression in Tfh cells, at age 2, in Bangladeshi children. The response to PMA/Ionomycin stimulation was quantified by the percentage of CD40L+ or CD57+ Tfh cells after stimulation divided by their percentage before stimulation. Response to stimulation was then adjusted for a log scale distribution. (**A**) Variable importance plots generated by random forest model, ranking predictors of CD40L and CD57 response to stimulation (CD40L on the top left and CD57 on the bottom left). Variables are ranked by importance, with significant predictors determined by GEE model and marked by asterisks. (**B**) Partial dependency plots depict the relationship between key environmental factors [e.g., days since last last antibiotic treatment, a total number of antibiotic treatments since day 2, Weight-for-height Z-score (WHZ), and diarrheal episodes] and Tfh cell response, with CD40L response on the left *y*-axis (pink) and CD57 response on the right y-axis (blue). Statistical significance was calculated by the GEE model and denoted by asterisks (*P < 0.05, **P < 0.01, ***P < 0.001, ****P < 0.0001).

Notable predictors identified included days since last antibiotic treatments, total number of antibiotic treatments by age 2, total number diarrheal episodes by age 2, and malnutrition scores such as weight-for-height Z-score (WHZ) (Fig. 3A). Partial dependency plots (Fig. 3B) further highlighted inverse relationships between these environmental factors and Tfh cell marker expression. Children who experienced fewer antibiotic treatments, fewer diarrheal episodes, and better WHZ scores were more likely to upregulate CD40L expression upon stimulation. Conversely, children with more frequent antibiotic use, more diarrheal episodes, and poorer malnutrition scores were associated with increased CD57 expression. Additionally, children who had not taken an antibiotic treatment in the last 2 weeks were more likely to have had an increase in both CD40L and CD57 expression. These findings suggested that early environmental exposures may influence Tfh cell functional programming, toward either an activation-supportive phenotype (CD40L+) or an exhaustion-prone phenotype (CD57+), with potential implications for immune competency and disease susceptibility in later life.

### Functional T follicular helper cell responses are correlated with the ability to mount an antibody response after vaccination or infection

To evaluate the relationship between Tfh cell functionality and downstream immune responses, we examined antibody concentration following vaccination or *Cryptosporidium* infection and correlated the increase of CD40L and CD57 expression on Tfh cells post-stimulation. *Cryptosporidium*, an endemic parasitic infection in Bangladesh, presented a relevant model for assessing pathogen-specific immune responses in this population.

**Fig 4:**
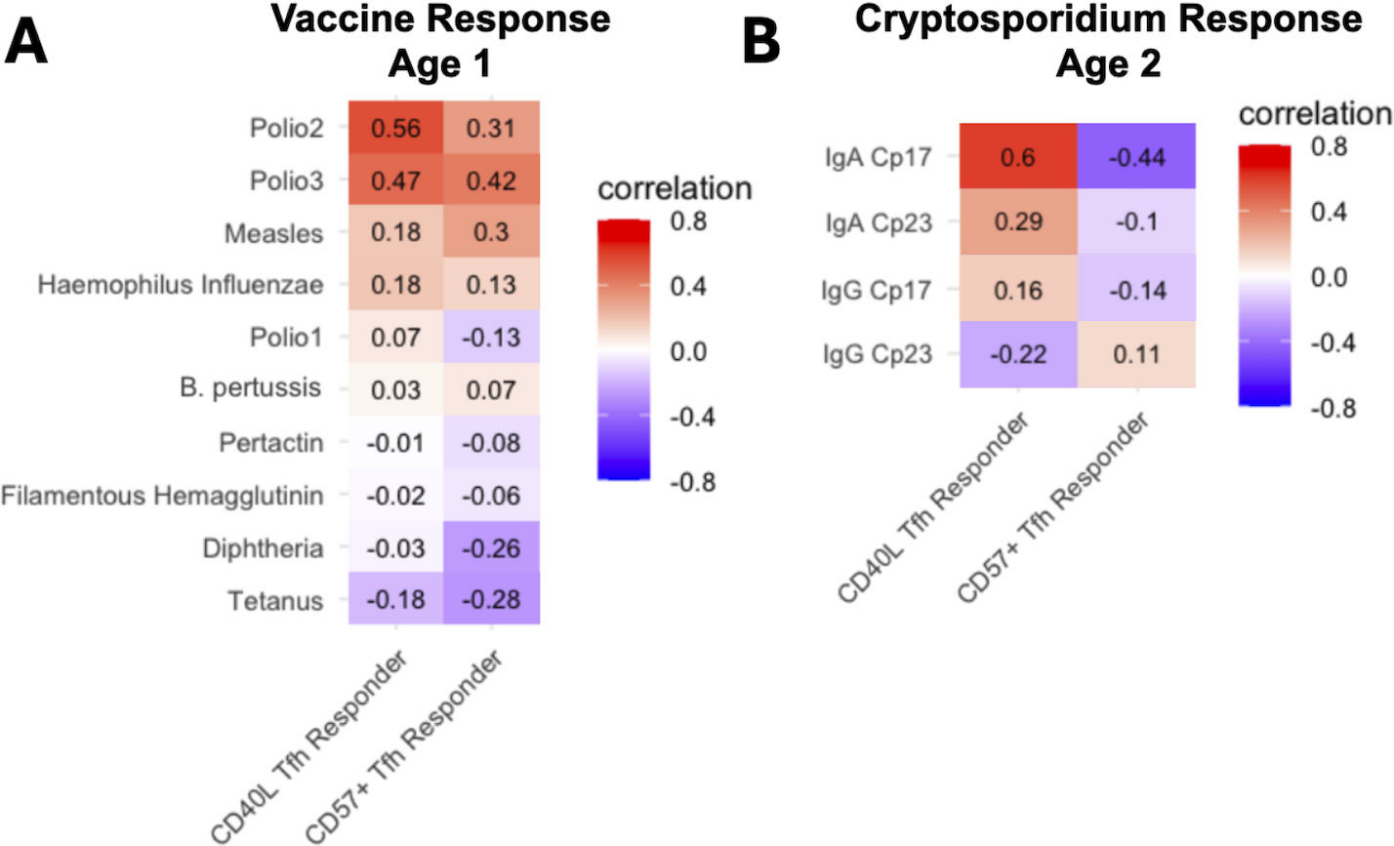
Correlation of Tfh cell functional response with antibody responses to vaccination and *Cryptosporidium* infection. (**A**) Heatmap displaying correlation coefficients between Tfh cell CD40L and CD57 response to stimulation and antibody titers against vaccine antigens. (**B**) Heatmap displaying correlation of CD40L and CD57 response to stimulation with IgA and IgG responses against *Cryptosporidium* sporozoite proteins Cp17 and Cp23.

Correlational analysis revealed that increased expression of CD40L and CD57 on Tfh cells in response to PMA/ionomycin stimulation was associated with higher antibody titers against certain vaccine antigens, specifically type 2 and type 3 poliovirus (Fig. 4A; Fig. S3). These findings implied that the ability of Tfh cells to upregulate activation and senescence markers may have been most necessary to support robust antibody responses following polio vaccination specifically. Additionally, we observed a significant positive correlation between high concentrations of IgA antibodies against the *Cryptosporidium* sporozoite protein Cp17 and CD40L expression. This indicated that Tfh cells expressing higher levels of CD40L may have played a role in promoting protective mucosal immunity against *Cryptosporidium*. Conversely, IgA levels against Cp17 were negatively correlated with CD57 expression, which suggested that Tfh cells with lower CD57 expression may have been more functionally active in mediating responses to parasitic infections (Fig. 4B).

## DISCUSSION

This study demonstrates the significant impact of early-life environmental exposures on T follicular helper (Tfh) cell functionality in Bangladeshi children, with important implications for immune competency and humoral immunity. By focusing on CD40L and CD57 (markers of Tfh cell activation and senescence), we reveal age-specific and environment-driven alterations in Tfh cell responses that may compromise immune support for B cell-mediated antibody production.

At age 2, some Bangladeshi children exhibit impaired CD40L upregulation and a significant reduction in CD57 expression upon stimulation. These findings suggest an exhaustion-like phenotype in Tfh cells that is not observed in American children of the same age. CD40L is critical for promoting B cell activation and germinal center formation, and impaired upregulation could hinder the generation of high-affinity antibodies and immunological memory. Meanwhile, the decreased CD57 expression upon stimulation may reflect accelerated apoptotic turnover of this population of Tfh cells due to chronic immune activation (16). However, these findings vary between individual children, suggesting that environmental factors may be influencing the functional ability of Tfh cell response to stimulation.

The PROVIDE study, which collected data from Bangladeshi children, initially enrolled 700 participants. To address the concern that study results may have been influenced by a biased withdrawal of the sickest children, we compared malnutrition scores (WAZ, HAZ, and WHZ) at the time of termination across all categories of withdrawal, including voluntary withdrawal, relocation, loss of contact, and death. The analysis showed no significant differences in malnutrition scores between groups, suggesting that the decision to leave the study was not disproportionately associated with poorer nutritional status. This result minimizes the likelihood of systematic bias affecting the outcomes and supports the robustness of our findings. While the subset of samples analyzed here represents a fraction of the original cohort, the consistency in malnutrition scores across termination groups provides additional confidence that the reported differences in Tfh cell functionality are reflective of the cohort as a whole and not artifacts of sample loss or selective attrition.

Our analysis identified key environmental factors that predict Tfh cell functionality upon stimulation. Days since the last antibiotic treatment, the number of antibiotic treatments, total diarrheal episodes, and malnutrition scores (WHZ) emerged as significant predictors of Tfh cell responses. Children with fewer antibiotic treatments, higher WHZ scores, and fewer diarrheal episodes were more likely to upregulate CD40L, indicative of an activation-supportive phenotype. Conversely, frequent antibiotic use, poor nutritional outcomes, and recurrent diarrheal episodes were associated with increased CD57 expression, which signifies association between senescence or impaired Tfh cell functionality and chronic environmental stressors.

The shapes of the Partial Dependency Plots provide important insights into the relationships between environmental factors and Tfh cell functionality. For example, significant changes in CD40L and CD57 responses were observed around a WHZ score of −1, near the clinical cutoff for defining malnutrition. This suggests a potential threshold effect, where moderate to severe malnutrition drives Tfh cells toward an exhaustion-like phenotype characterized by increased CD57 expression and reduced CD40L upregulation. Conversely, children with WHZ scores above this threshold exhibited more activation-supportive CD40L responses, underscoring the critical role of nutritional status in shaping immune function. Notably, the sharp increase in CD57 expression with greater antibiotic exposure and recurrent diarrheal episodes may reflect cumulative immune stress, leading to an exhaustion-prone phenotype. However, the effect of antibiotic exposure is limited to 2 weeks since the most recent treatment, with both the ability to increase CD40L and CD57 restored past this time point. While this finding was not expected, it is important to note that the temporal relation of antibiotic treatments was only identified as a significant predictor for CD40L expression. The direct effect of antibiotics on Tfh cell function has not been extensively studied. However, dysbiosis of the gut microbiome has been associated with altered Tfh cell populations, including both impaired and overactivated responses, as seen in autoimmune conditions and experimental models (17, 18). These findings underscore the influence of early-life exposures on immune programming and highlight potential modifiable factors to improve immune health in high-risk populations. Interventions targeted to optimize immune development in resource-limited settings, such as improving sanitation to reduce diarrheal burden, may promote Tfh cell function and potentially influence the ability to mount protective humoral responses post vaccination and infection.

In addition to examining environmental predictors, we investigated the downstream effects of Tfh cell functionality on humoral immunity. CD40L expression was positively correlated with IgA responses to the *Cryptosporidium* sporozoite antigen Cp17, indicating that Tfh cells with an activation-supportive phenotype may be important for effective immune responses. Conversely, CD57 expression was negatively correlated with anti-Cp17 IgA levels, supporting the hypothesis that a senescent or exhausted Tfh cell phenotype may be less effective in promoting humoral immunity. The positive association between CD40L expression and *Cryptosporidium*-specific IgA levels highlights the role of activation-supportive Tfh cells in mucosal immunity, particularly in environments with high pathogen exposure.

However, a different pattern emerged when examining the relationship between Tfh cell marker expression and antibody titers against vaccine antigens. Both CD40L and CD57 expression were positively correlated with increased antibody concentrations for Polio Type 2 and Type 3 antigens. This is most likely the result of the standard EPI vaccine administration in the first year of life. At age 1, Tfh cell response to stimulation was more homogenous in the Bangladeshi children, representing a healthier phenotype. It is possible that the results may have been more like that of the *Cryptosporidium* antibody response, in relation to CD40L and CD57 inverse correlation, if administered in the second year of life, when Tfh cells had an exhaustion-like phenotype. However, at age 1, Tfh cell function may not yet have been fully shaped by environmental exposures to the extent observed at age 2. As a result, increased CD57 expression upon stimulation at this earlier time point may not negatively impact the ability to mount a robust antibody response, contrasting with the patterns observed later in development.

CD40L has been previously established as an important factor in immunity against *Cryptosporidium*, particularly in patients with CD40L deficiency, who are at significantly increased risk of cryptosporidiosis (19–21). This underscores the importance of CD40L in promoting effective immune responses to parasitic infections. Our findings, which highlight the impaired CD40L upregulation in Bangladeshi children, align with this understanding and further suggest that environmental factors may exacerbate susceptibility to *Cryptosporidium* through diminished Tfh cell functionality.

A key limitation of this study is that while data was collected longitudinally, the analysis did not exclusively use paired samples from the same children at each timepoint. As a result, the ability to model true within-individual longitudinal changes is constrained, and the findings represent cross-sectional analyses of distinct subsets of children at each age. Nevertheless, the consistency of observed trends in CD40L and CD57 expression across multiple years supports the generalizability of these findings to the broader cohort. To more robustly assess the longitudinal development of Tfh cell functionality and its determinants, future studies should prioritize designs that ensure serial sampling of the same individuals over time.

Although restricted in sample size, this study highlights the significant role of early immune programming in shaping long-term immune outcomes. The potential impact of an exhaustion-prone Tfh phenotype on susceptibility to infectious diseases and vaccine efficacy underscores the need for further research. These findings deepen our understanding of how environmental factors influence Tfh cell functionality and immunity and emphasize the importance of addressing environmental disparities to promote optimal immune health in children globally. Future research should focus on interventions to mitigate these environmental effects, such as targeted nutritional supplementation and improved access to healthcare, to enhance immune competence during critical windows of immune system development.

Additionally, the results emphasize Tfh cell functional markers, particularly CD40L and CD57, serve as valuable indicators of effective antibody-mediated immunity in response to vaccination and endemic infections. The distinct correlation patterns observed for CD40L and CD57 reinforce their roles in modulating the immune response and should continue to be studied, with CD40L upregulation associated with enhanced antibody production, while increased CD57 expression signaling a shift toward cellular senescence or diminished antibody support. These findings pave the way for leveraging Tfh cell markers to assess and potentially improve immune responses in populations at high risk for infectious diseases.

## MATERIALS AND METHODS

### Data acquisition and analysis

This study utilized data from two cohorts: PROVIDE and STORK.

- PROVIDE Study: Based in Mirpur, Dhaka, Bangladesh, this cohort consisted of 700 children from a lower socioeconomic area with high microbial exposure. After a door-to-door population census of the community was done to identify pregnant women, children were enrolled within 7 days of birth and monitored biweekly, by at-home visits. At each visit, questionnaires collected data on diarrheal illnesses, antibiotic use, breastfeeding practices (exclusive and non-exclusive), and nutritional status. Whole blood samples were collected annually up to age 4. At study enrollment, mothers completed a lifestyle questionnaire. Vaccinations, including polio (types 1, 2, and 3), *Haemophilus influenzae* type B (Hib), tetanus, diphtheria, and acellular pertussis, were administered before age 1, while the measles vaccine was administered at 65 weeks of age (22).
- STORK Study: Based in the San Francisco Bay Area, California, this cohort included children from a low socioeconomic area with low microbial exposure. Pregnant mothers (majority Hispanic) were recruited primarily from public clinics before 36 weeks of gestation and interviewed weekly post-birth, via phone call or email. Whole blood samples were collected annually up to age 3. Blood samples collected within 15 weeks of a child’s birthday were grouped together for analysis. For example, samples collected between 37–67 weeks of age were categorized as year 1 (23).

Peripheral blood mononuclear cells (PBMCs) were isolated using conventional methods previously described (12). Cryopreserved PBMCs from both studies were thawed, stimulated non-specifically with PMA/Ionomycin for 6 hours, and stained with metal-conjugated antibodies. Data acquisition was performed on a CyTOF2 mass cytometry instrument following established protocols. Detailed methodology for CyTOF immunophenotyping and analysis has been documented in detail (12), and the corresponding data files are publicly accessible through the Flow Repository database (experiment ID: FR-FCM-ZYV8).

The data analyzed in this study were obtained from the Flow Repository database. While the PROVIDE study followed children longitudinally from birth to age four, this analysis used cross-sectional data, with subsets of children contributing samples at each time point rather than paired samples from the same individuals across ages (Table 1). This approach maximized the use of the data set but limits the ability to model within-individual longitudinal changes. Statistical models treated each time point as independent and paired unstimulated and stimulated samples were analyzed for each child at each time point.

**TABLE 1:** Samples used for analysis

Study	Total sample #	18 weeks	52 weeks	104 weeks	156 weeks	208 weeks
PROVIDE	75	14	26	23	6	6
STORK	22	**–^*a*^**	5	9	8	**–**

^*a*^ – indicates time points at which no samples were available.

### Data processing

CD4+ T cells were gated manually from the CD3+ T cell population following the exclusion of any dead cells, B cells, monocytes, and natural killer cells. Samples with fewer than a count of 400 CD4+ T cells were excluded from further analysis. Additionally, samples lacking matched untreated and stimulated groups were removed, ensuring that only paired untreated and stimulated samples were included in the final analysis. T follicular helper (Tfh) cells were gated from the CD4+ T cell population and defined as CXCR5+ PD-1+, CD4+ T cells. From the total Tfh cell population, CD40L+ and CD57+ Tfh cells were further gated out of the total Tfh cell population. The proportion of CD40L+ or CD57+ Tfh cells was calculated by dividing the count of CD40L+ or CD57+ Tfh cells by the total Tfh cell count within each sample, separately for untreated and stimulated groups. The Tfh cell response to stimulation was defined as the ratio of marker expression in the stimulated group to the untreated group. This ratio was calculated as the proportion of CD40L+ or CD57+ Tfh cells in the stimulated sample divided by the corresponding proportion in the untreated sample, providing a quantitative measure of Tfh functional response to stimulation.

### Prediction modeling and statistics

Random forest feature importance evaluation was used to identify key environmental factors influencing Tfh cell functionality. Input variables included environmental factors such as exclusive and total breastfeeding duration, number of antibiotic treatments, nutritional metrics (WAZ, HAZ, and WHZ scores), number of diarrheal episodes, CRP levels, annual family income, and parental education levels. The outcome variables were the log-transformed ratios of CD40L+ and CD57+ Tfh cells as a ratio of stimulated to unstimulated conditions. Models were trained using 10-fold cross-validation with tree counts of 100, 300, 500, and 700, and a minimum node sizes ranging from 1 to 9. Through the grid search, the optimal tuning parameters are selected to fit the random forest modeling. Variable importance was evaluated based on the mean decrease in the Gini index, which quantifies the predictive contribution of each variable.

Generalized estimating equations (GEE) were subsequently employed to model associations between the top five predictors identified by random forest analysis. The log-transformed ratio of stimulated to unstimulated cell counts for CD40L+ and CD57+ Tfh cells (as a proportion of all Tfh cells) served as the response variable. GEEs accounted for the repeated measures and clustering inherent in the data, providing robust estimates of significant associations between environmental factors and Tfh cell functional outcomes. Statistical analyses were performed using appropriate software with significance thresholds set at *P* < 0.1.

### Antibody quantification

Antibody concentrations post-vaccination for measles, *Bordetella pertussis* toxin, filamentous hemagglutinin, pertactin, diphtheria toxoid, tetanus, and *Haemophilus influenzae* type B antigens were quantified using standardized assays, reported as IU/mL, EU/mL, or ng/mL. IgA and IgG concentrations against *Cryptosporidium* sporozoite antigens Cp17 and Cp23 were measured using enzyme-linked immunosorbent assays (ELISA).

For ELISA, microtiter plates were coated with antigen solution and incubated overnight at 4°C. Plates were then washed, and a blocking buffer was applied to minimize nonspecific binding. Plasma samples were added to the wells and incubated, followed by another blocking buffer application. Subsequently, HRP-conjugated anti-human secondary antibodies were added to detect bound primary antibodies. TMB (3,3′,5,5′-tetramethylbenzidine) substrate solution was then added for color development, and the reaction was stopped with a stop solution. Absorbance was measured at 450 nm to quantify antibody levels. All measurements were performed in triplicate to ensure reproducibility.

## Data Availability

The data set is available under experiment ID FR-FCM-ZYV8 in Flow Repository (http://flowrepository.org/id/FR-FCM-ZYV8).

## SUPPLEMENTAL MATERIAL

Supplemental Figures mbio.03980-24-s0001.pdf Figures S1 to S3.

ASM does not own the copyrights to Supplemental Material that may be linked to, or accessed through, an article. The authors have granted ASM a non-exclusive, world-wide license to publish the Supplemental Material files. Please contact the corresponding author directly for reuse.
