# Erratum for Van Fossen et al., “Influence of environmental exposures on T follicular helper cell function and implications on immunity: a comparison of Bangladeshi and American children”

**DOI:** 10.1128/mbio.01435-25

**Published:** 2025-06-10

**Authors:** Dana M. Van Fossen, Hyunjae Cho, Lisa E. Wagar, Jennie Z. Ma, Julie Parsonnet, Rashidul Haque, Mark M. Davis, William A. Petri

## ERRATUM

Volume 16, no. 4, e03980-24, 2025, https://doi.org/10.1128/mbio.03980-24. Page 1, byline: The affiliation number for Hyunjae Cho should be 7.

Page 10, Author Affiliations: Affiliation 7 should be added as follows: Department of Statistics, University of Virginia, Charlottesville, USA.

