# Supplementary material for: Influence of environmental exposures on T follicular helper cell function and implications on immunity: a comparison of Bangladeshi and American children
Source: mBio. 2025 Mar 10;16(4):e03980-24. doi: 10.1128/mbio.03980-24 (PMC11980363; doi:10.1128/mbio.03980-24)
Supplement: Supplemental Figures — Figures S1 to S3. [file mbio.03980-24-s0001.pdf]

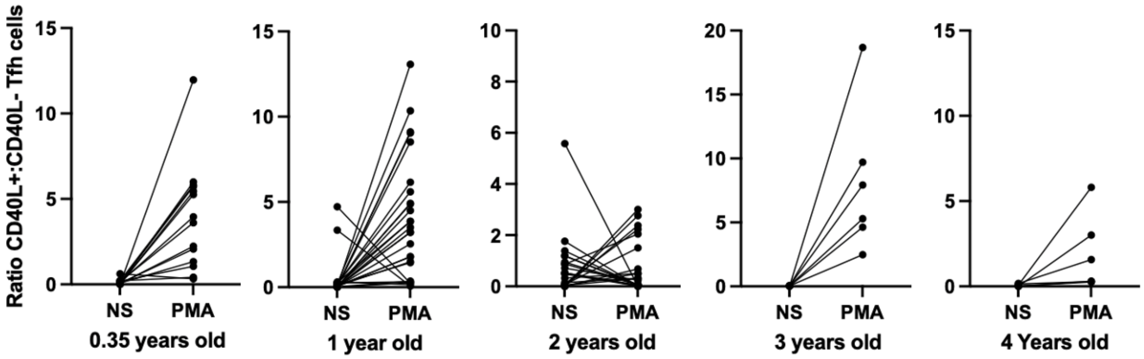

Supplemental Figure 1: CD40L expression in Tfh cells following PMA/Ionomycin stimulation across age groups. Ratio of CD40L+ to CD40L- T follicular helper (Tfh) cells in non-stimulated (NS) and PMA/Ionomycin-stimulated (PMA) conditions across different age groups (0.35, 1, 2, 3, and 4 years). Each panel corresponds to a specific age group, with paired data from individual children connected by lines. The y-axis shows the ratio of CD40L+ to CD40L- Tfh cells.

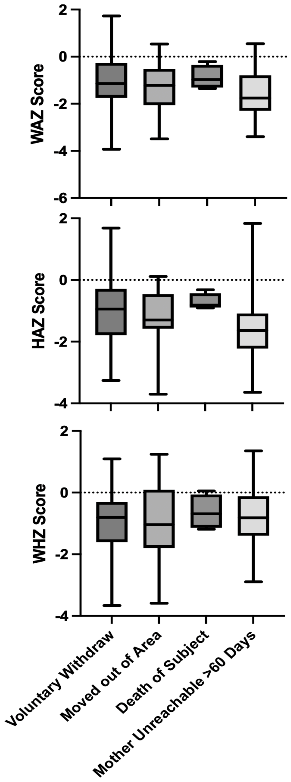

Supplemental Figure 2: Comparison of malnutrition scores (WAZ, HAZ, WHZ) across termination categories of the PROVIDE study. Box plots comparing malnutrition scores at the time of termination across different termination categories: voluntary withdrawal (n=54), moved out of study area (n=23), death of subject (n=4), and mother unreachable >60 days (n=23). **Top Panel**: Weight-for-Age Z-scores (WAZ). **Middle Panel**: Height-for-Age Z-scores (HAZ). **Bottom Panel**: Weight-for-Height Z-scores (WHZ). No significant differences were observed among the groups for

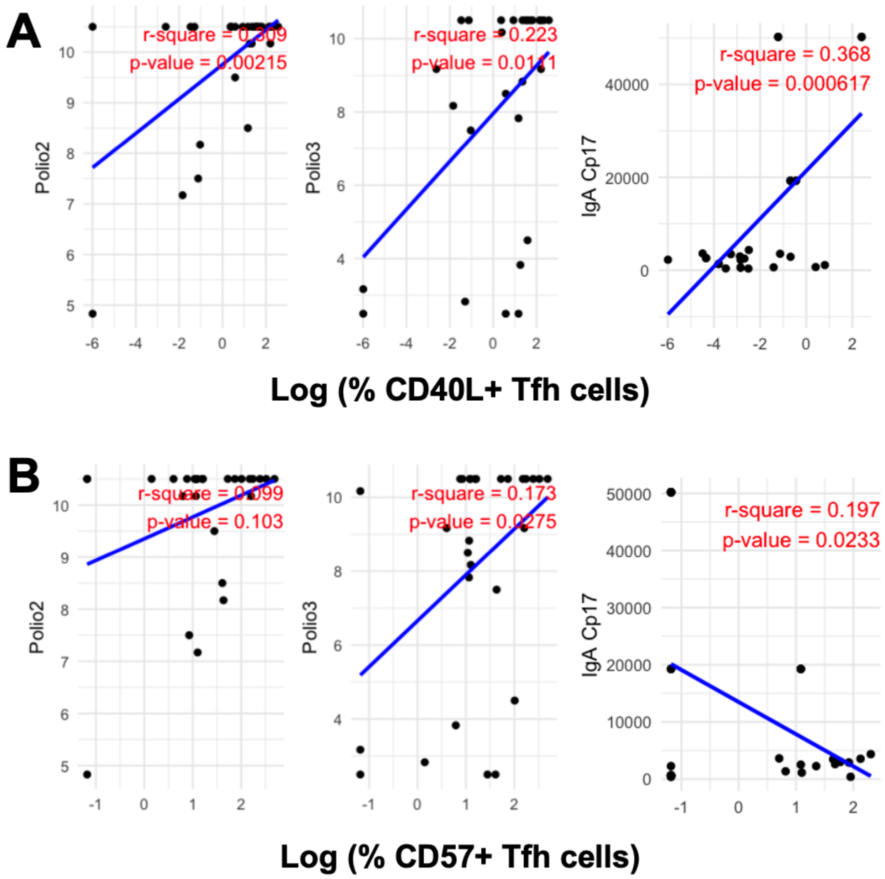

Supplemental Figure 3: Correlation of CD40L+ and CD57+ Tfh cells with top antibody responses post vaccination and Cryptosporidium infection. (A) Scatter plots showing the correlation between the log-transformed percentage of CD40L+ T follicular helper (Tfh) cells and antibody titers for Poliovirus Type 2 (left), Poliovirus Type 3 (middle), and IgA concentrations against Cryptosporidium Cp17 (right). Blue lines represent linear regression models. The r-squared values and p-values are indicated in red. (B) Scatter plots showing the correlation between the log-transformed percentage of CD57+ Tfh cells and antibody titers for Poliovirus Type 2 (left), Poliovirus Type 3 (middle), and IgA concentrations against Cryptosporidium Cp17 (right). Blue lines represent linear regression models.
